# Genome-Wide Identification and Phylogenetic Analysis of *TRP* Gene Family Members in Saurian

**DOI:** 10.3390/ani12243593

**Published:** 2022-12-19

**Authors:** Lin Zhang, Ning Li, Buddhi Dayananda, Lihu Wang, Huimin Chen, Yunpeng Cao

**Affiliations:** 1 School of Health and Nursing, Wuchang University of Technology, Wuhan 430223, China; 2 State Key Laboratory of Microbial Technology, Institute of Microbial Technology, Shandong University, Qingdao 266237, China; 3 College of Food Science, Nanjing Xiaozhuang University, Nanjing 211171, China; 4 School of Agriculture and Food Sciences, The University of Queensland, Brisbane, QLD 4072, Australia; 5 School of Landscape and Ecological Engineering, Hebei University of Engineering, Handan 056038, China; 6 School of Basic Medical Sciences, Hubei University of Chinese Medicine, Wuhan 430065, China; 7 CAS Key Laboratory of Plant Germplasm Enhancement and Specialty Agriculture, Wuhan Botanical Garden, Chinese Academy of Sciences, Wuhan 430074, China

**Keywords:** *TRP* gene family, saurian, evolution, genome-wide, thermal sensors

## Abstract

**Simple Summary:**

A total of 251 putative *TRP*s from saurian are divided into 2 groups, belonging to 6 *TRP*s subfamilies, excluding the *TRPN* subfamily. The most conserved proteins of *TRP* box 1 are located in motif 1, and those of *TRP* box 2 are located in motif 10. The *TRPA* and *TRPV* in saurian tend to be one cluster, as a sister cluster with *TRPC*, and the *TRPM* is a root of group I. *TRPM*, *TRPV,* and *TRPP* are clustered into two clades, and *TRPP* is organized into *TRP PKD1-*like and *PKD2-*like. Segmental duplications mainly occur in the *TRPM* subfamily, and the tandem duplications only occur in the *TRPV* subfamily. Fifteen sites were under positive selection for *TRPA1* and *TRPV2* genes. The branch model revealed that positive selection fit the data better than the null model for the genes *TRPC5* and *TRPV3*.

**Abstract:**

The transient receptor potential plays a critical role in the sensory nervous systems of vertebrates in response to various mechanisms and stimuli, such as environmental temperature. We studied the physiological adaptive evolution of the TRP gene in the saurian family and performed a comprehensive analysis to identify the evolution of the thermo-TRPs channels. All 251 putative TRPs were divided into 6 subfamilies, except TRPN, from the 8 saurian genomes. Multiple characteristics of these genes were analyzed. The results showed that the most conserved proteins of *TRP* box 1 were located in motif 1, and those of *TRP* box 2 were located in motif 10. The *TRPA* and *TRPV* in saurian tend to be one cluster, as a sister cluster with *TRPC*, and the *TRPM* is the root of group I. The *TRPM*, *TRPV*, and *TRPP* were clustered into two clades, and *TRPP* were organized into *TRP PKD1-*like and *PKD2-*like. Segmental duplications mainly occurred in the *TRPM* subfamily, and tandem duplications only occurred in the *TRPV* subfamily. There were 15 sites to be under positive selection for *TRPA1* and *TRPV2* genes. In summary, gene structure, chromosomal location, gene duplication, synteny analysis, and selective pressure at the molecular level provided some new evidence for genetic adaptation to the environment. This result provides a basis for identifying and classifying *TRP* genes and contributes to further elucidating their potential function in thermal sensors.

## 1. Introduction

Ectotherms’ body temperature, an important physiological parameter, is essential for the optimal performance of physiological functions within a narrow thermal environment [1,2,3,4]. The capacity to maintain body temperature directly indicates the fitness of individuals [5]. Changes in ambient temperature influence the individuals’ physiology, performance, and fitness [6]. The thermally heterogeneous changes, including microhabitats, seasonality, and climate change, influence individuals’ body temperature [2]. Individuals employ behavioral or postural adjustments to control their body temperature to avoid harmful extremes [7]. The interactions between ecological and physiological traits directly determine thermal performance, which is the result of selective pressures and evolution [1,8,9]. Changes in ambient temperature influence the individual physiology, altering performance and fitness. To balance the trade-off of the heat exchange between the individuals and their thermal environment, individuals have to evolve some reliable thermosensory proteins, to rapidly respond with physiology or behavior to the complex spatial and temporal changes in the thermal environment [10]. The ability to sense environmental and internal temperatures is a prerequisite for the evolution of thermoregulation [11]. Hence, individuals require a sophisticated physiological system to sense the ambient temperature for survival [12]. 

Ectotherms employed sensory neurons in the peripheral nervous system, as temperature-sensitive ion channels [13]. Transient receptor potential (TRPs) consists of Ca^2+^ permeable non-selective cation channels, which function in numerous physiological processes and homeostatic functions [14]. This response is due to a signaling cascade that produces a transitory change in receptor potential [15,16]. The TRPs were divided into seven subfamilies based on their amino acid sequences and structures, as TRPA (Ankyrin), TRPC (Canonical), TRPM (Melastatin), TRPML (Mucolipin), TRPN (Nompc), TRPP (Polycystin), and TRPV (Vanilloid) [17]. Depending on the variations in the luminal/extracellular domain between transmembrane helix 1 (S1) and S2 [18,19], the seven subfamilies are recognized and divided into group I (TRPA/C/M/N/V) and group II (TRPML and TRPP). The TRPP subfamily is an ancient member of the TRPs [19]. During the evolution of the TRPs, six subfamilies have been observed in vertebrates [20], except TRPN, which only occurs in zebrafish and invertebrates [21]. The thermo-TRPs, of the TRP channels activated by temperature, are divided into heat-sensitive proteins (TRPV1-4) [22] and cold-sensitive proteins (TRPM8, TRPC5, and TRPA1) [23,24,25]. The TRPV1, a classic thermo-TRP, is directly activated by high temperatures (≥ 43 °C) in humans [26]. The TRPM8 regulates thermoregulation as it relates to cold temperature sensation in lizards because it does not participate in regulating warm temperature behaviors such as gaping [2]. To adapt to thermal niches, some changes in TRP improve thermal perception and responses in the individual’s large-scale evolution. All these findings indicate the important roles of TRP channels in the different environmental stimuli. However, to date, most of the research on TRP proteins has mostly been performed on mammals. 

In reptiles, TRPs play a key role in interpreting thermal stimuli to rapidly and accurately sense the environment around them [11,13,27,28]. Inhibition of *TRPV1* and *TRPM8* by the blocker capsazepine in *Crocodylus porosus* abolished the typical ectotherms shuttling behavior and led to significantly altered body temperature patterns [11]. It indicates that the function of TRPs in reptiles for thermoregulation is similar to that in mammals. In *Takydromus tachydromoides*, cold treatment reduced *TRPV4* expression in the brain, tongue, heart, lungs, and muscles in the hibernation species, but levels of *TRPV4* mRNA in the skin remained unaffected after entering hibernation and cold treatment [29]. In *Mauremys reevesii*, the embryos moved toward a mild heat source when the ambient temperature was above 29 °C due to TRPA1 activation, but embryos moved away from the noxious heat source when the ambient temperature was above 33 °C due to TRPV1 activation [30]. *Thermophis baileyi* exhibited species-specific temperature-sensing molecular strategies (amino acid replacements) in the *TRPA1,* which did not influence the temperature-response margin but increased the heat sensitivity [31]. Moreover, previous studies have shown that thermosensitive gating in a given channel is species-specific, and multiple channels act together to sense the thermal environment [10]. However, there is little information on the TRP proteins’ gene family in reptiles. 

In this study, to test the physiological adaptive evolution of the TRP gene family in saurian, we performed a comprehensive analysis of the genome sequence data of eight saurian species and identified the TRP protein gene family. We analyzed their phylogenetic relationships, conserved motifs, gene structure, and gene duplication. To improve the understanding of the evolution of the thermo-TRP channels in saurian, we performed selective pressure analysis on thermo-TRPs to identify the positive selection. 

## 2. Materials and Methods

### 2.1. Identification of TRP Gene Family in Saurian

We downloaded the saurian genomes (Table S1), including those of *Anolis carolinensis*, *Gekko japonicus*, *Lacerta agilis*, *Podarcis muralis*, *Pogona vitticeps*, *Sceloporus undulatus*, *Sphaerodactylus townsendi,* and *Zootoca vivipara*, from the NCBI database (https://www.ncbi.nlm.nih.gov/genome, accessed on 22 July 2022). We employed TBtools [32] to generate the Hidden Markov Model (HMM) of TRPs based on *Homo sapiens* from HGNC (https://www.genenames.org, accessed on 22 July 2022), *Xenpus tropicalis* from Xenbase (https://www.xenbase.org/entry/, accessed on 22 July 2022), and *Danio rerio* from ZFIN (https://www.zfin.org/, accessed on 22 July 2022) to identify the TRP members based on the HMM of TRPs by using HMMER3 software [33]. To avoid missing probable TRP members, we used a BLASTp algorithm-based search using molluscan TRPs amino acid sequences as queries with a cutoff e-value ≤ 1e-5 [34]. Overlapping genes were manually removed. All candidate TRP genes were filtered by using the NCBI Conserved Domain Database (CDD, https://www.ncbi.nlm.nih.gov/Structure/cdd/cdd.shtml, accessed on 22 July 2022) [35,36] and Multiple EM for the Motif Elicitation (MEME) online tool (http://meme-suite.org/tools/meme, accessed on 22 July 2022) [37]. The MEME was set with the following parameters: any number of repetitions, a maximum of 10 motifs, and an optimum motif width of 6–50 residues [38], and MEME online was employed to analyze the motif structure of TRP proteins. The Gene Structure View (Advanced) was employed for the visualization of conversed domains and motifs in TBtools [32]. Next, using TBtools, we detected the exact chromosomal locations of all TRP genes through a BLAST search of the genome sequences. Due to the association with the highly conserved region of 23–25 amino acids C-terminal to the transmembrane domains [39], we ran a comparative analysis of the conserved domain in saurian.

### 2.2. Phylogenetic Analysis

The TRPs were aligned using MAFFT [40] and implemented in PhyloSuite [41]. According to the best model as implemented in IQ-TREE2, the maximum likelihood (ML) tree was computed and built with a bootstrap test (5000 replicates) and the SH-aLRT test (1000 random addition replicates) [42]. Phylogenetic consensus trees were edited by using iTOL (https://itol.embl.de, accessed on 22 August 2022) [43].

### 2.3. Chromosome Locations and Synteny Analysis

The chromosomal locations of TRP genes, except for *Gekko japonicus* and *Pogona vitticeps,* were obtained from general feature format files. Gene location visualization from the GFF was used to map the distribution of TRP genes. To identify the orthologous genes among eight saurian genomes, we used OrthoFinder [44], and MCSanX was employed to conduct colinearity analyses of all TRPs between and within species [45]. Circos was used to visualize the colinear relationships of TRPs [46] and the distribution of these genes on the chromosomes in TBtools.

### 2.4. Selective Pressure Analysis

Based on the previous research [12,47], we selected seven genes, including TRPA1, TRPM8, TRPC5, and TRPV1-4, to analyze the selection pressure in the eight saurian genomes, and then calculated the proportions of the non-synonymous (*d*N)/synonymous (*d*S) evolutionary rate (ω) using EasyCodeML v1.12 [48] to represent the selective selection. Different ω values indicated a different type of selection: ω > 1 shows the positive selection, ω = 1 represents the neutral selection, and ω < 1 represents the purifying selection [49]. We employed a fast unconstrained Bayesian approximation (FUBAR) on the Datamonkey website to detect the positive selection implemented [50]. The analysis was calculated based on the site model (M8a vs. M8) [51] and branch model (two ratios vs. one ratio) with the likelihood ratio test (LRT) threshold of *p *< 0.05, elucidating adaptation signatures within the genome. Amino acid sites under positive selection were detected using Bayesian empirical bayes (BEB) inference, with an 80% posterior probability cutoff [52].

## 3. Results

### 3.1. TRP Genes in Saurian

**Figure 1:**
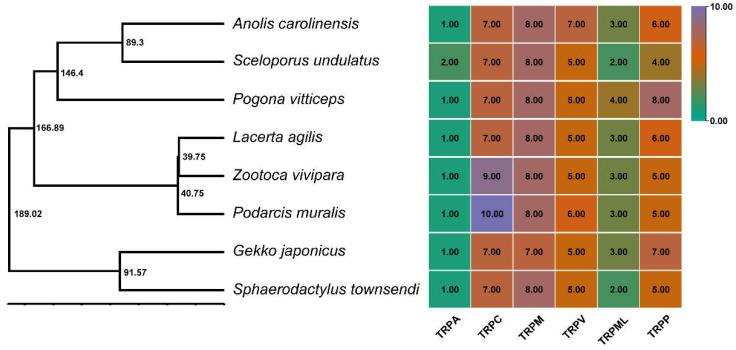
Species tree of eight saurian and *TRP* numbers of each species. The time tree of the eight saurian was built by the TIMETREE 5 (**left**, the number is the divergence time), and the numbers of the *TRP* family detected in each species were listed accordingly (**right**).

A total of 32, 30, 30, 33, 27, 28, 28, and 31 TRPs were identified in *A. carolinensis*, *G. japonicus*, *L. agilis*, *P. muralis*, *P. vitticeps*, *S. undulatus*, *S. townsendi,* and *Z. vivipara*, respectively (Figure 1 and Table S1). Ten conserved motifs were identified using MEME in saurian (Figure S1). Motif 2 was present in each saurian TRP protein, and the TRPM included all 10 motifs. No TRPN proteins were found in the eight saurian genomes (Figure 1). The most conserved proteins of TRP box 1 were located in motif 1, and those of TRP box 2 were located in motif 10.

### 3.2. Phylogenetic Relationships of TRPs in Saurian Genomes

**Figure 2:**
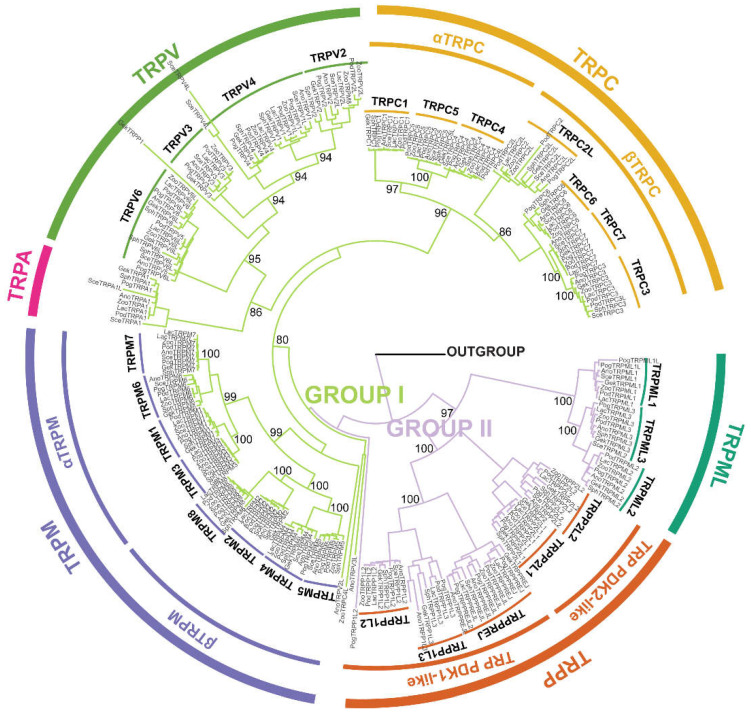
Analysis of the maximum likelihood (ML) tree of *TRP*s in saurian. Moderate green and grayish violet represent group I and group II, respectively.

To explore the evolutionary differences and origins of these TRP protein families in saurian, we further analyzed the molecular histories of these genes. We performed maximum-likelihood (ML) analysis on the amino acid sequences of all 251 TRPs using the ML method with 1000 bootstrap replicates (Figure 2). The *TRP*s were clustered into six subgroups (TRPA, TRPC, TRPP, TRPM, TRPML, and TRPV), which belong to two monophyletic clades as group I and group II. Group I contained four subfamilies, *TRPA*, *TRPC*, *TRPM,* and *TRPV*, and group II contained two subfamilies, *TRML* and *TRPP*, respectively. Phylogenetic analysis indicated that *TRPA* and *TRPV* in saurian tended to be one cluster, as the sister cluster with *TRPC*, and *TRPM* is the root of group I. The TRPM, TRPV, and TRPP proteins clustered into two clades (Figure 2) and TRPM proteins contained the αTRPM clade (including *TRPM3, TRPM1, TRPM6,* and *TRPM7*) and the βTRPM clade (including *TRPM4, TRPM5, TRPM2,* and *TRPM8*). *TRPV1*, *TRPV2*, *TRPV3,* and *TRPV4* belonged to TRPV protein group I, and the TRPV protein group II contained TRPV5 protein and TRPV6 proteins, which were annotated as TRPV 5/6. TRPP was organized into two clades: TRPP1-like (including *TRPPREJ*, *TRPP1L2,* and *TRPP1L3*) and TRPP2-like (including TRPP2, TRPP2L1, and TRPP2L2).

### 3.3. Syntenic Analysis of TRPs in Saurian Genomes

**Figure 3:**
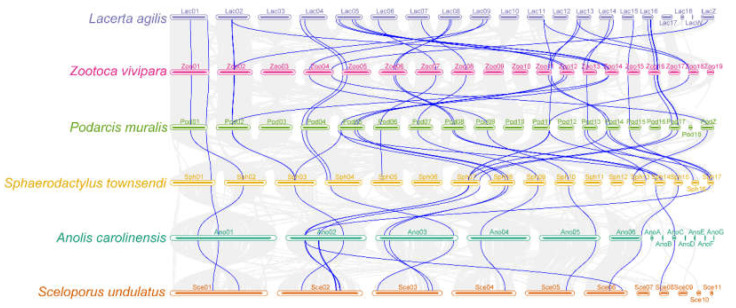
Synteny analysis of *TRP* genes among inter-genomes of saurian. The gray line in the background shows colinear blocks in the six saurian genomes, and the blue line highlights colinear *TRP* pairs.

**Figure 4:**
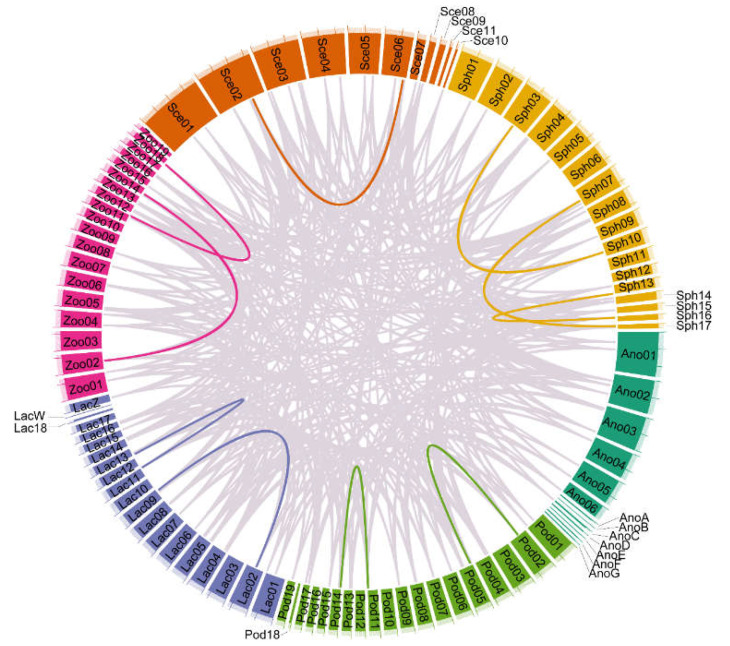
Colinear region of the *TRP* gene of saurian. The gray lines represent all the colinear blocks in *TRP* gene pairs among species, while the highlight-colored lines represent *TRP* gene pairs subjected to segmental duplication within species. Chromosome numbers are shown at the bottom of each chromosome.

To further identify the orthologous relationships and evolutionary origins of *TRP*s in saurian, we identified 737 orthologous gene pairs (Table S3). We investigated the synteny among six saurian genomes with chromosome-level genomes using MCScanX, and the data showed that high-level microsynteny was maintained among the saurian genomes (Figure 3). There was a one-to-one correspondence between the gene lineages and syntenic orthologous groups (Figure S2). The *TRP*s from saurian contributed at least one *TRP* to each subfamily. In our study, most members of the *TRP* family were distributed on chromosomes (Figure S3 and Table S4). Based on the results of the syntenic analysis, the segmental duplications (SD) and tandem duplications (TD) were the *TRP* duplication types in all genomes (Figure 4), but the *TRP*s did not experience duplication events in *A. carolinensis* (Figure 4). The SD mainly occurred in the *TRPM* subfamily, and TD only occurred in the *TRPV* subfamily. There was only one SD in *S. undulatus* (rna-XM_042476030.1 vs. rna-XM_042451064.1), two SD in *L*. *agilis* (rna-XM_033163651.1 vs. rna-XM_033166883.1 and rna-XM_033160619.1 vs. rna-XM_033140165.1), *P*. *muralis* (rna-XM_028749274.1 vs. rna-XM_028706666.1 and rna-XM_028728955.1 vs. rna-XM_28718097.1), and *Z. vivipara* (rna-XM_035131044.1 vs. rna-XM_035099594.1 and rna-XM_035137878.1 vs. rna-XM_035104957.1), and three SD in *S*. *townsendi* (rna-XM_048513883.1 vs. rna-XM_048519142.1, rna-XM_048508742.1 vs. rna-XM_048487239.1, and rna-XM_048519624.1 vs. rna-XM_048503482.1), respectively (Figure S4). There was only one TD in *S. townsendi* (rna-XM_048504402.1 vs. rna-XM_048504571.1), two TD in *S*. *undulatus* (rna-XM_042442354.1 vs. rna-XM_042442355.1 and rna-XM_042447702.1 vs. rna-XM_042452285.1), three TD in *P. muralis* (rna-XM_028708247.1 vs. rna-XM_028708248.1, rna-XM_028708530.1 vs. rna-XM_028708708.1, and rna-XM_028712426.1 vs. rna-XM_028712789.1) and *Z. vivipara* (rna-XM_035141029.1 vs. rna-XM_035097275.1, rna-XM_035139054.1 vs. rna-XM_035139047.1, and rna-XM_035139316.1 vs. rna-XM_035139320.1), and four TD in *L. agilis* (rna-XM_033145813.1 vs. rna-XM_033145814.1, rna-XM_033171541.1 vs. rna-XM_033172593.1, rna-XM_033172595.1 vs. rna-XM_033171884.1, and rna-XM_033173257.1 vs. rna-XM_033173677.1), respectively.

### 3.4. Selective Pressure Analysis in Saurian TRP Gene

**Figure 5:**
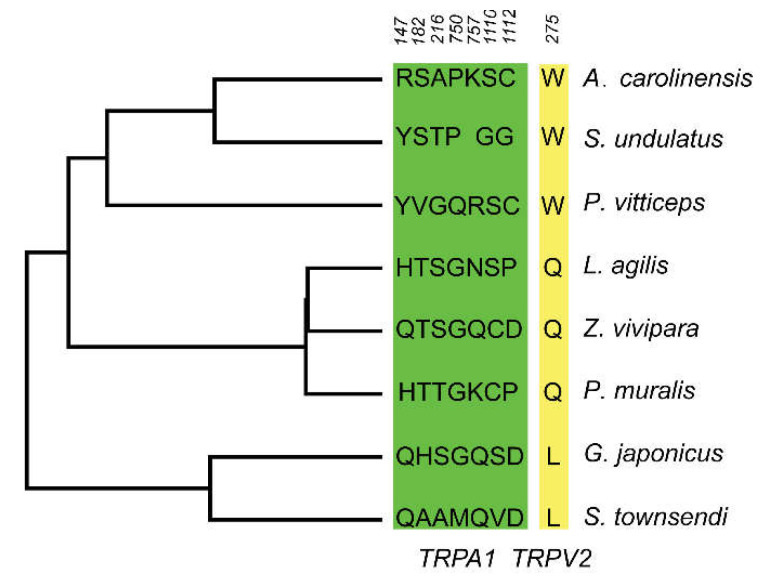
Amino acid changes in selected sites across the saurian phylogeny. Amino acid changes occurred in genes *TRPA1* (green) and *TRPV2* (yellow). The numbers represent the amino acid positions in the genes *TRPA1* and *TRPV2*.

To determine whether the individual codons in each gene were subjected to positive selection, we used the site model (M8a vs. M8) in the dataset. The results showed that the M8 model, which included positive selection, fit the data better than the neutral model M8a. Specifically, the M8 model detected 15 sites to be under positive selection at *TRPA1* and *TRPV2* genes (Table 1 and Figure 5), most of which were located in the coil of *TRPA1* and *TRPV2* genes. Significant evidence of positive selection was further identified using the FUBAR model implemented in Datamonkey. Additionally, FUBAR also identified 13 sites in these two genes under diversifying selection with a posterior probability > 0.8. 

A branch model with two ratios was used to explore whether saurian with daily activity rhythms (classified into two groups, i.e., diurnal and nocturnal saurian) evolved under different evolutionary pressures. The results revealed that the model that included positive selection fit the data better than their null model at genes *TRPC5* (two ratios vs. one ratio: *p* = 0.025) and *TRPV3* (two ratios vs. one ratio: *p* = 0.032) (Table 2).

## 4. Discussion

In this study, all 251 identified putative *TRPs* with 6 subfamilies, except *TRPN*, were identified from 8 saurian genomes. This study is the first study to characterize the repertoire and evolutionary patterns of the *TRP* gene family in saurian. All *TRP*s were highly conserved in their sequences and structural features. Based on the selective pressure analysis and the activity time of the species, we hypothesized that *TRPA1*, *TRPV2*, *TRPV3*, and *TRPC5* are the thermo-TRP channels of saurian. Our results provide a novel view of the saurian thermal sensor system at the molecular level.

Over the past two decades, more and more species were sequenced since the genome of *A. carolinensis* was reported, and genomic data provide us with convenient conditions for analyzing evolution, including gene structure, chromosome location, gene duplication, synteny analysis, and selective pressure. Genome-wide screening revealed 32, 30, 30, 33, 27, 28, 28, and 31 *TRP*s in *A. carolinensis*, *G. japonicus*, *L. agilis*, *P. muralis*, *P. vitticeps*, *S. undulatus*, *S. townsendi,* and *Z. vivipara*, respectively (Figure 1 and Table S2). The TRPs belonged to six *TRP* subfamilies, except for the *TRPN* subfamily. The *TRPN* members are only present in worms, flies, and zebrafish, except in Antarctic fish [10], mammals [39], and saurian. Reptiles share a common ancestor with mammals and have an important amniote phylogeny position [53]. The number of members of each *TRP* subfamily is relatively more stable in vertebrates than in invertebrates, but there are more *TRPP*-like genes in saurian (Figure 1, right). We found a highly conserved motif named motif 2 in all saurian *TRP* genes. The most conserved portions of the TRP domain were identified as the TRP boxes 1 and 2 [39], and the results indicated that boxes 1 and 2 were located in motif 1 and motif 10, respectively. 

The results showed that the complement of TRPs in saurian was similar to that in mammals. The finding supports those of recent analyses of the evolutionary history of TRP. In the phylogenetic analysis, we restructured the initial reliability phylogenetic relationship with the high topology consistency of *TRP*s, and further divided these *TRPs* into two groups, which supported the previous results in the literature that members of the *TRP*s are divided into group I and group II [10,54], and the *TRPP* is located at the root of the ML tree, supporting the *TRPP* subfamily as the ancestor of *TRP*, where the members of the TRPP subfamily extend from yeast to mammals [19]. TRPM proteins are divided into the *αTRPM* and *βTRPM*. The *βTRPM* clade contains the *TRPM2*, *TRPM4*, *TRPM5,* and *TRPM8*, where the *TRPM2*, *TRPM4,* and *TRPM5* are activated by heat, but the *TRPM8* responds to cold temperatures [55]. TRPV proteins belong to two groups that assist with this function, TRPV1-4 proteins are defined as thermosensitive [46], and TRPV5/6 proteins’ function is to maintain Ca^2+^ homeostasis [47]. In lizards, TRPP channels are organized into PKD1-like and PKD2-like, except for the brivido subfamily, which differs from the previous studies [54,56].

Moreover, the syntenic analysis showed the number of homology pairs within and/or among species, but the *TRP*s have not experienced the duplication event in *A. carolinensis*. Gene duplication events, such as large-scale duplication (whole-genome duplication or segmental duplication) and tandem duplication, are the main drivers for the generation of novel genes. Large-scale duplication events play a key role in gene family evolution, and tandem duplication does not increase the number of conserved genes [57]. In saurian, the SD mainly occurs in the *TRPM* subfamily, but the TD only occurs in the *TRPV* subfamily. TRPM and TRPV proteins clustered into two clades, which appears to be due to the ancestral saurian genome duplication, and the event is still observed in fish [10]. The number of duplicated gene pairs in *TRP*s (from 3 to 6, including SD and TD) did not correlate with the genome size and chromosome number in saurian (Figure S3). It is similar in the genome size and chromosome number in saurian, but the number of colinear *TRP* gene pairs varied among species, associated with the phylogenetic site. 

The vulnerability of lizards to climate warming depends on the sensitivity of the individuals to temperature variation [7], which increases the risk of population decline and extinction [58]. However, the diel activity pattern reflects the capacity of adaptation to the variable environment [59], in which the ambient temperature at night is lower than that during the daytime. The ancestral state of geckos is nocturnal, except for *Phelsum* and *Lygodactylus* in Gekkonidae and Sphaerodactylidae species [60]. Hence, most geckos have lower body temperatures than diurnal species [61,62], reducing the metabolic rate and metabolic by-products as well as oxidative damage [63]. In this study, the selection analysis showed significant evidence of positive selection, including *TRPA1*, *TRPC5*, *TRPV2,* and *TRPV3* in saurian (Table 1 and Table 2). In vertebrates, *TRPV1*’s physiological role in sensing noxious high temperatures is well-conserved among vertebrate species [12]. *TRPC5* and *TRPM8* were identified as cold-relative genes [24,64]. In lizard (*Takydromus tachydromoides*) and snake (*Elaphe quadrivirgata*), *TRPV4* in the skin may act as an environmental temperature sensor throughout the reptilian lifecycle [32]. In *C. porosus*, hot-sensing *TRPV1* and cold-sensing *TRPM8* have the potential to act as internal and external temperature sensors, respectively [11]. 

## 5. Conclusions

In this study, all 251 putative *TRPs* were divided into 6 subfamilies, except *TRPN*, from 8 saurian genomes, such as *A. carolinensis*, *G. japonicus*, *L. agilis*, *P. muralis*, *P. vitticeps*, *S. undulatus*, *S. townsendi,* and *Z. vivipara*. The results provided a comprehensive analysis of the subfamily classification, gene structure, chromosomal location, gene duplication, synteny analysis, and selective pressure of TRPs in saurian and provided new evidence for the physiological adaptive evolution of environmental changes. This investigation provides a basis for identifying and classifying TRP genes and contributes to elucidating their potential function in thermal sensors, facilitating future functional characterization of thermo-TRPs and providing important clues for saurian thermal sensors.

## Figures and Tables

**Table 1:** Positive selection at amino acid sites of saurian *TRP*s.

	PAML			Datamonkey
	−2ΔlnL	Site Model	ω Value	FUBAR
*TRPA1*	10.58	4, 17, 147, 182, 216, 750, 757, 828, 873, 1110*, 1112*	3.18	92, 115, 147, 174, 182*, 216*, 232, 750, 757, 1110*, 1112
*TRPV2*	6.28	275, 715, 717*, 735*	4.35	38, 275, 563

Note: Positively selected sites inferred by both methods are underlined. *: Selective pressure > 0.9.

**Table 2:** Log likelihood and omega values estimated under the branch model of *Gekko japonicus* on *TRP* genes.

	Model	−lnL	−2ΔlnL	*p*-Value	ω Value	
					Background	Foreground
*TRPC5*	Two ratios	−8267.20	4.99	0.025	0.03	0.07
	One ratio	−8269.69				
*TRPV3*	Two ratios	−7736.71	4.62	0.032	0.06	0.11
	One ratio	−7739.02				

## Data Availability

Publicly available datasets were analyzed in this study. This data can be found here: *Anolis carolinensis* [AnoCar2.0]*, Gekko japonicus* [Gekko_japonicus_V1.1]*, Lacerta agilis* [rLacAgi1.pri]*, Podarcis muralis* [PodMur_1.0]*, Pogona vitticeps* [pvi1.1]*, Sceloporus undulatus* [SceUnd_v1.1]*, Sphaerodactylus townsendi* [MPM_Stown_v2.3]*, and Zootoca vivipara* [UG_Zviv_1)].

## Supplementary Materials

The following supporting information can be downloaded at: https://www.mdpi.com/article/10.3390/ani12243593/s1, Figure S1: Phylogenetic relationships, gene structure, and motif distributions of saurian *TRP* genes. Figure S2: Analysis of ML tree and orthologous gene pairs among the saurian. The different color links suggested orthologous gene relationships of different saurian. Figure S3: Distribution of *TRP* gene pairs within species. The gray lines represent all the colinear blocks in gene pairs within species, while the red lines represent *TRP* gene pairs subjected to segmental duplication within species. Chromosome numbers are shown at the bottom of each chromosome. Table S1: List of saurian genomes and *TRP* gene sequences information in this study. Table S2: The link between gene ID and gene symbol. Table S3: Duplicated *TRP* gene pairs in saurian. Table S4: The divergence between duplicated *TRP* gene pairs in saurian.
