# Supplementary figures and images for: Genome-Wide Identification and Phylogenetic Analysis of TRP Gene Family Members in Saurian
Source: Animals (Basel). 2022 Dec 19;12(24):3593. doi: 10.3390/ani12243593 (PMC9774356; doi:10.3390/ani12243593)

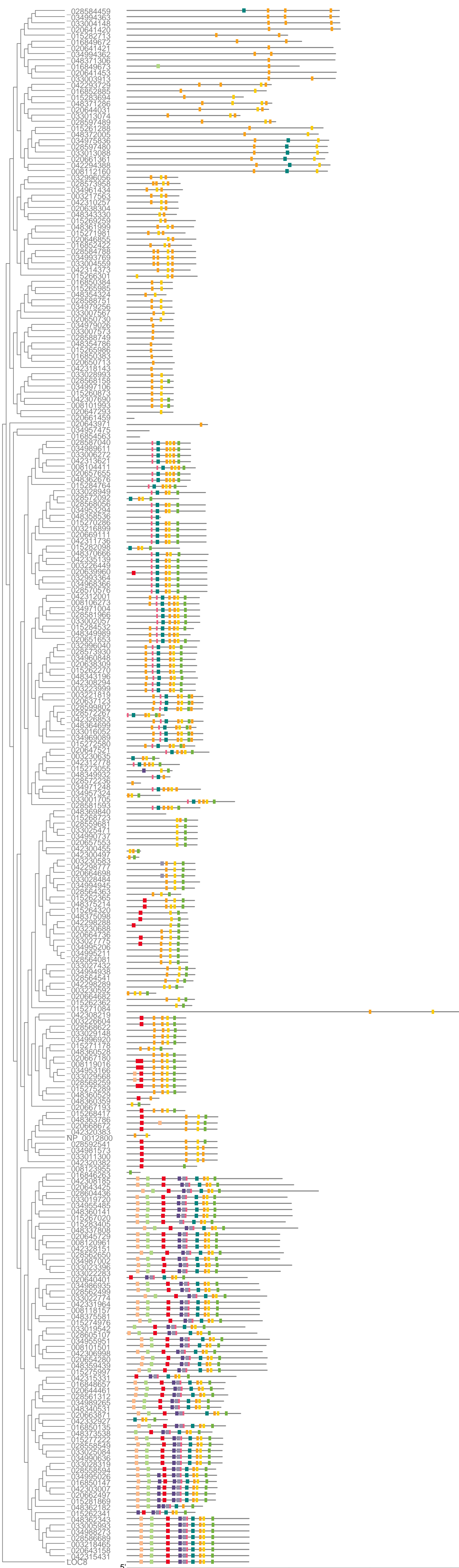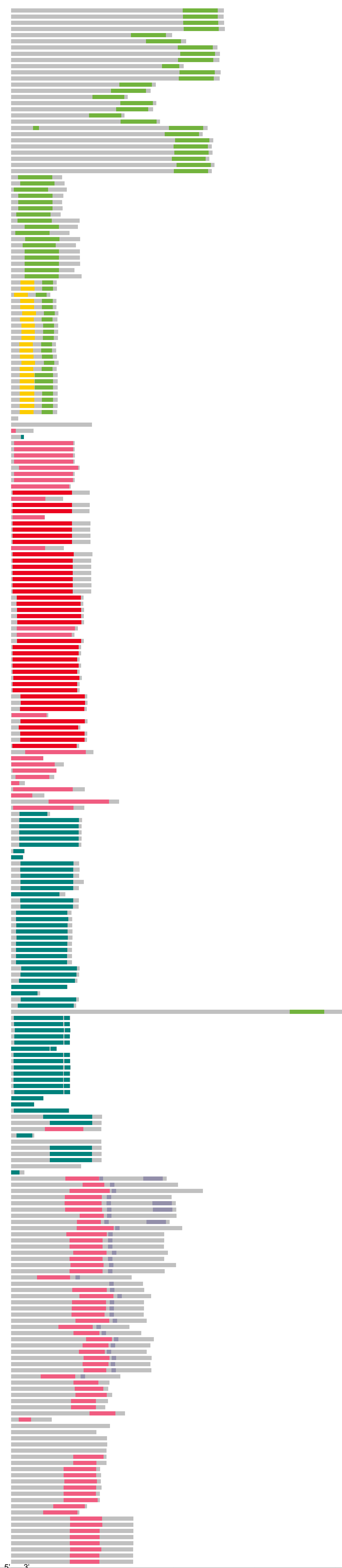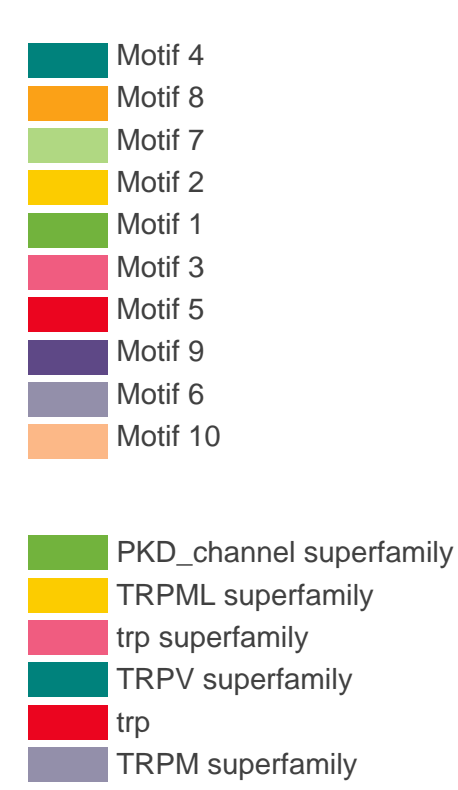

Supplement: Supplementary file 1 [file animals-12-03593-s001.zip › Figure S1.pdf]

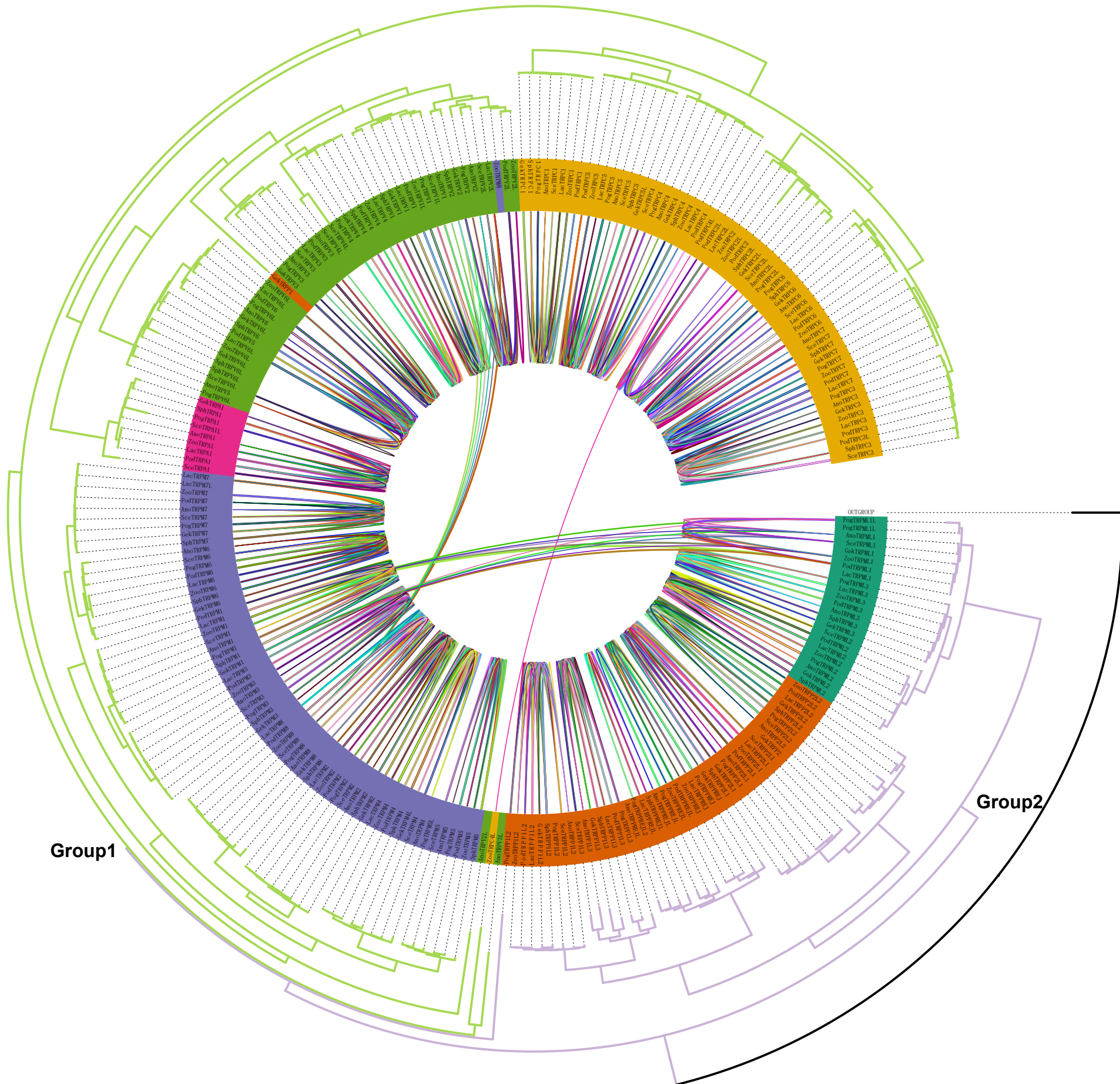

Supplement: Supplementary file 1 [file animals-12-03593-s001.zip › Figure S2.pdf]
