# Supplementary material for: Genome-Wide Identification and Phylogenetic Analysis of TRP Gene Family Members in Saurian
Source: Animals (Basel). 2022 Dec 19;12(24):3593. doi: 10.3390/ani12243593 (PMC9774356; doi:10.3390/ani12243593)
Supplement: Supplementary file 1 [file Figure S3.pdf › Figure S3.pdf]

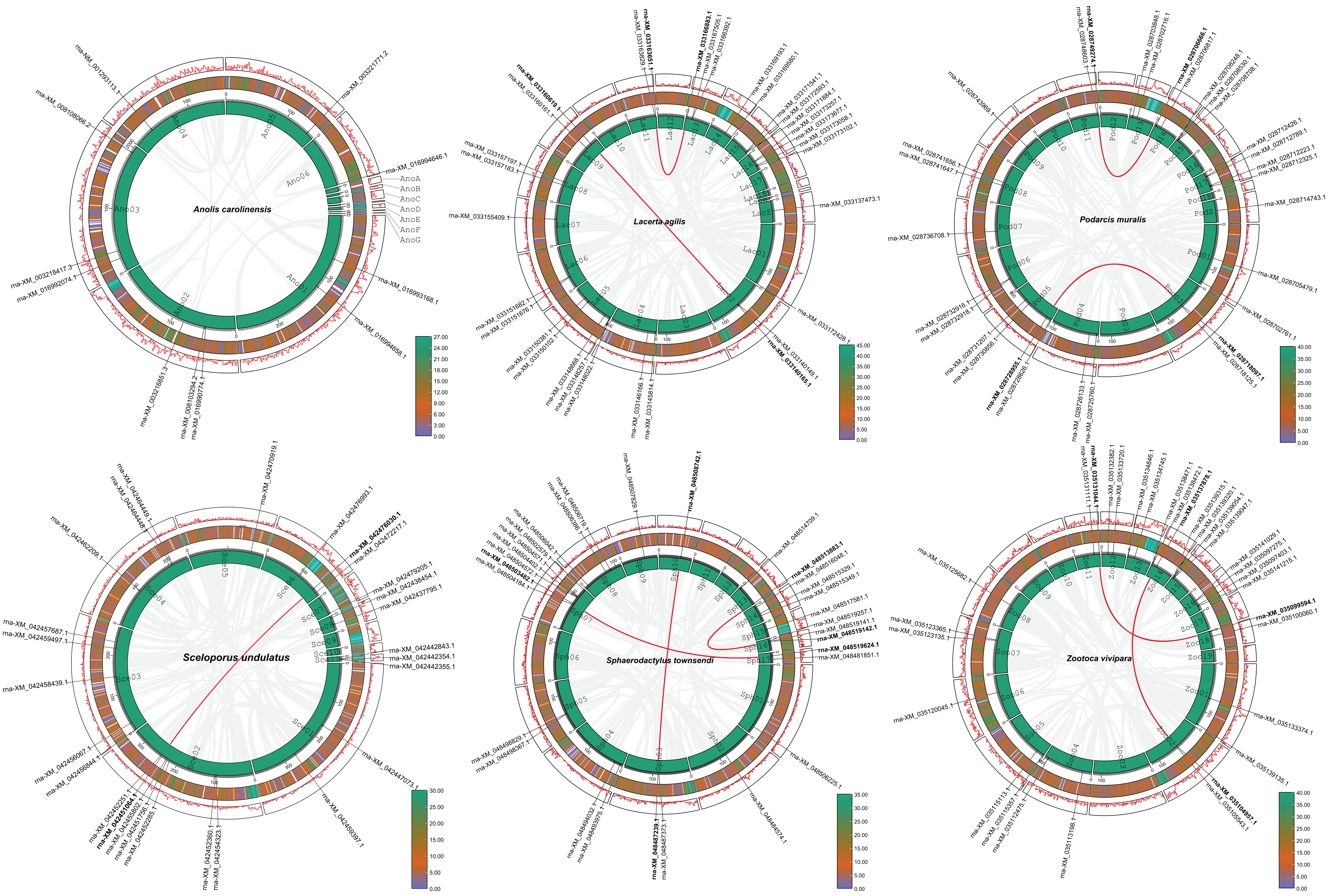

Anolis carolinensis
Ano01
Ano02
Ano03
Ano04
Ano05
Ano06
AnoA
AnoB
AnoC
AnoD
AnoE
AnoF
AnoG
rna-NM_001293113.1
rna-XM_003221771.2
rna-XM_008108066.2
rna-XM_016994646.1
rna-XM_003218417.3
rna-XM_016992074.1
rna-XM_016993168.1
rna-XM_016994658.1
rna-XM_003216851.3
rna-XM_008103294.2
rna-XM_016990774.1
27.00
24.00
21.00
18.00
15.00
12.00
9.00
6.00
3.00
0.00
Lacerta agilis
rna-XM_033160619.1
rna-XM_033160161.1
rna-XM_033157197.1
rna-XM_033157183.1
rna-XM_033155409.1
rna-XM_033151682.1
rna-XM_033151676.1
rna-XM_033150381.1
rna-XM_033150102.1
rna-XM_033148668.1
rna-XM_033148257.1
rna-XM_033148022.1
rna-XM_033146166.1
rna-XM_033145814.1
rna-XM_033140149.1
rna-XM_033140165.1
rna-XM_033172428.1
rna-XM_033137473.1
rna-XM_033163651.1
rna-XM_033163829.1
rna-XM_033166883.1
rna-XM_033167505.1
rna-XM_033166392.1
rna-XM_033169193.1
rna-XM_033169560.1
rna-XM_033171541.1
rna-XM_033172593.1
rna-XM_033171884.1
rna-XM_033173257.1
rna-XM_033173677.1
rna-XM_033173058.1
rna-XM_033173102.1
45.00
40.00
35.00
30.00
25.00
20.00
15.00
10.00
5.00
0.00
Podarcis muralis
rna-XM_028743969.1
rna-XM_028741656.1
rna-XM_028741647.1
rna-XM_028736708.1
rna-XM_028732916.1
rna-XM_028732918.1
rna-XM_028731207.1
rna-XM_028730856.1
rna-XM_028728955.1
rna-XM_028728626.1
rna-XM_028726133.1
rna-XM_028725760.1
rna-XM_028718097.1
rna-XM_028718125.1
rna-XM_028702761.1
rna-XM_028705479.1
rna-XM_028714743.1
rna-XM_028712426.1
rna-XM_028712789.1
rna-XM_028712223.1
rna-XM_028712325.1
rna-XM_028708248.1
rna-XM_028708530.1
rna-XM_028708708.1
rna-XM_028706666.1
rna-XM_028706817.1
rna-XM_028703848.1
rna-XM_028702716.1
rna-XM_028749274.1
rna-XM_028748603.1
40.00
35.00
30.00
25.00
20.00
15.00
10.00
5.00
0.00
Sceloporus undulatus
rna-XM_042462209.1
rna-XM_042464449.1
rna-XM_042464445.1
rna-XM_042470919.1
rna-XM_042476993.1
rna-XM_042476030.1
rna-XM_042472217.1
rna-XM_042479205.1
rna-XM_042438454.1
rna-XM_042437795.1
rna-XM_042442843.1
rna-XM_042442354.1
rna-XM_042442355.1
rna-XM_042457687.1
rna-XM_042459497.1
rna-XM_042458439.1
rna-XM_042456067.1
rna-XM_042456844.1
rna-XM_042452251.1
rna-XM_042451064.1
rna-XM_042455802.1
rna-XM_042451756.1
rna-XM_042452285.1
rna-XM_042452360.1
rna-XM_042454323.1
rna-XM_042459397.1
rna-XM_042447073.1
30.00
25.00
20.00
15.00
10.00
5.00
0.00
Sphaerodactylus townsendi
rna-XM_048507829.1
rna-XM_048508742.1
rna-XM_048514709.1
rna-XM_048513883.1
rna-XM_048516048.1
rna-XM_048515329.1
rna-XM_048515349.1
rna-XM_048517581.1
rna-XM_048519257.1
rna-XM_048519141.1
rna-XM_048519142.1
rna-XM_048519624.1
rna-XM_048481851.1
rna-XM_048506225.1
rna-XM_048484574.1
rna-XM_048487239.1
rna-XM_048487373.1
rna-XM_048494032.1
rna-XM_048493975.1
rna-XM_048498829.1
rna-XM_048498367.1
rna-XM_048504184.1
rna-XM_048503482.1
35.00
30.00
25.00
20.00
15.00
10.00
5.00
0.00
Zootoca vivipara
rna-XM_035125682.1
rna-XM_035123365.1
rna-XM_035123135.1
rna-XM_035120045.1
rna-XM_035115113.1
rna-XM_035115357.1
rna-XM_035112475.1
rna-XM_035113198.1
rna-XM_035104957.1
rna-XM_035105543.1
rna-XM_035139135.1
rna-XM_035133374.1
rna-XM_035099594.1
rna-XM_035100060.1
rna-XM_035141029.1
rna-XM_035097275.1
rna-XM_035097403.1
rna-XM_035141215.1
rna-XM_035131044.1
rna-XM_035131111.1
rna-XM_035132382.1
rna-XM_035133720.1
rna-XM_035134846.1
rna-XM_035134745.1
rna-XM_035137878.1
40.00
35.00
30.00
25.00
20.00
15.00
10.00
5.00
0.00
